# A Novel Coloration of Polyester Fabric through Green Silver Nanoparticles (G-AgNPs@PET)

**DOI:** 10.3390/nano9040569

**Published:** 2019-04-08

**Authors:** K. M. Faridul Hasan, Md. Nahid Pervez, Md. Eman Talukder, Mst. Zakia Sultana, Sakil Mahmud, Md. Mostakim Meraz, Vipul Bansal, Cao Genyang

**Affiliations:** 1 State Key Laboratory of New Textile Materials and Advanced Processing Technologies, Wuhan Textile University, Wuhan 430200, China; 2 School of Chemistry and Chemical Engineering, Wuhan Textile University, Wuhan 430200, China; nahid.tex92@gmail.com (M.N.P.); zakia1136@gmail.com (M.Z.S.); 3 Guangzhou Institute of Advanced Technology, Chinese Academy of Sciences, Nansha, Guangzhou 511458, China; 2654410096@mails.ucas.ac.cn; 4 Ningbo Institute of Material Technology and Engineering, Chinese Academy of Sciences, Ningbo 315201, China; sakilhabib@gmail.com; 5 College of Chemical and Chemistry Engineering, Xiamen University, Xiamen 361005, China; 20420171155788@stu.xmu.edu.cn; 6 Sir Ian Potter NanoBioSensing Facility, NanoBiotechnology Research Laboratory, School of Science, RMIT University, Melbourne, VIC 3000, Australia; vipul.bansal@rmit.edu.au

**Keywords:** green silver nanoparticles, polyester, chitosan, coloration, sustainable textile

## Abstract

This paper reports a novel route for the coloration of polyester fabric with green synthesized silver nanoparticles (G-AgNPs@PET) using chitosan as a natural eco-friendly reductant. The formation of AgNPs was confirmed by UV-visible spectroscopy. The morphologies and average particles size of G-AgNPs was investigated by transmission electron microscope (TEM) analysis. The uniform deposition of G-AgNPs on the PET fabric surface was confirmed with scanning electron microscopy (SEM) and Fourier transform infrared (FT-IR) spectroscopy. The thermal properties were investigated using a thermogravimetric analyzer (TGA). The coloration and fastness properties of fabric were found to be significantly improved, a result related to the surface plasmon resonance of G-AgNPs. The antibacterial properties of fabric were also found to be excellent as more than 80% bacterial reduction was noticed even after 10 washing cycles. Overall, the proposed coating process using green nanoparticles can contribute to low-cost production of sustainable textiles.

## 1. Introduction

Textile coloration can be summarized as the relatively simple application of colorant(s) to a substrate in a solution medium. The coloring process typically involves a number of operations including dyeing, pigmenting and printing [1] and as a result releases large amounts of contaminated colored wastewater, which is considered to be a significant environmental pollutant due to its recalcitrant chemical nature (e.g., dyes/pigments) [2,3]. Many efforts are ongoing regarding the establishment of green textile coloration in terms of cost effectiveness and producing harmless non-contaminant materials associated with the subsequent process [4,5,6]. In recent years, the introduction of nanotechnology in the textile coloration process has provided a viable option to meet the current scenario [7,8,9].

Nanoparticles have received significant attention as an emerging class of colorant for textiles. Compared to conventional synthetic or natural dyes, nanoparticles have no chromophore (responsible for color) but rather their color properties are meditated by shape and size [10]. Among these nanoparticles, the majority of this research has focused on silver nanoparticles (AgNPs) as a sustainable textile coloring agent [11,12,13] as well as determining their ultimate fate after discharge into the environment [14]. AgNPs show excellent color stability due to their distinctive localized surface plasmon resonance (LSPR) properties [15]. The significant connection between the morphology of silver nanoparticles and their color is exceptionally useful for their utilization in various textile finishing applications [16]. Control of the morphology of AgNPs is a promising method to tailor the LSPR band and successfully tune the color of silver nanoparticles. As indicated by the literature, there are three methodologies available for fabric coloration using nanoparticles: fabrics impregnated with a colloidal nanoparticles solution, formation of fabric/nanoparticle nanocomposites during the spinning process, and in situ synthesis of nanoparticles on fabric [17]. The functionalization of synthetic fabrics through AgNPs has received significant attention, particularly with respect to polyester-based fabrics [18,19]. Some research of note in this regard focuses on AgNP-treated polyester fabrics for improving their regular performance. For example, Radetic et al. [20] employed corona treatment (electrical discharge at atmospheric pressure) on polyester and polyamide textile materials to improve their bind interaction prior to application of silver nanoparticles. They found that both corona-pretreated fabrics had high laundering durability and significant antibacterial activity after modification of AgNPs. Gorensek et al. [21] further reported that AgNPs on Ar/N_2_ (50%:50%) plasma-pretreated polyester fabric demonstrated high dyeability and acceptable antibacterial properties against *P. aeruginosa*, *E. coli* and *S. faecalis*.

Up to now, numerous synthesis routes have been established for the preparation of AgNPs for textile coloration [22,23,24]. The majority of AgNPs synthesized have been found to be associated with hazardous chemicals and yield toxic organic byproducts. Many recent studies focus on the green synthesis of AgNPs for coloration of textiles with advanced applications for coloration [25,26]. In this context, chitosan may be a viable green stabilizing agent for AgNPs with remarkable antimicrobial properties against vegetative bacteria [27,28,29,30]. It is well known that chitosan is a polymeric compound that enables surface binding to nanoparticles with high antibacterial properties due to its functional groups [31]. Therefore, chitosan-mediated AgNPs could be a potential green coloration source for the textile industry.

The present study focused on developing chitosan-mediated green silver nanoparticles for polyester fabric coloration (G-AgNPs@PET) and, to the best of authors’ knowledge, presents such a focus for the first time in the literature. The characteristics of G-AgNPs in solution phase were studied by UV-visible spectrophotometer and Fourier transfer infrared spectroscopy (FTIR). Moreover, color characteristics and antibacterial properties of the treated fabrics were also evaluated. In sum, we developed a simple, green and feasible route to produce green coloration of polyester-based fabric for multifunctional applications. 

## 2. Materials and Methods

### 2.1. Materials

The 100% gray plain weave polyester (PET) fabric with 25 warps and 20 wefts in cm was supplied by Esquel Group (Gaoming, China). Silver nitrate (AgNO_3_) (99.98%), chitosan and ascorbic acid (99%) were purchased from the Sinopharm Chemical Reagent company limited (Shanghai, China). All chemicals were used without further purification.

### 2.2. Preparation and Coloration of G-AgNPs@PET

**Scheme 1:**
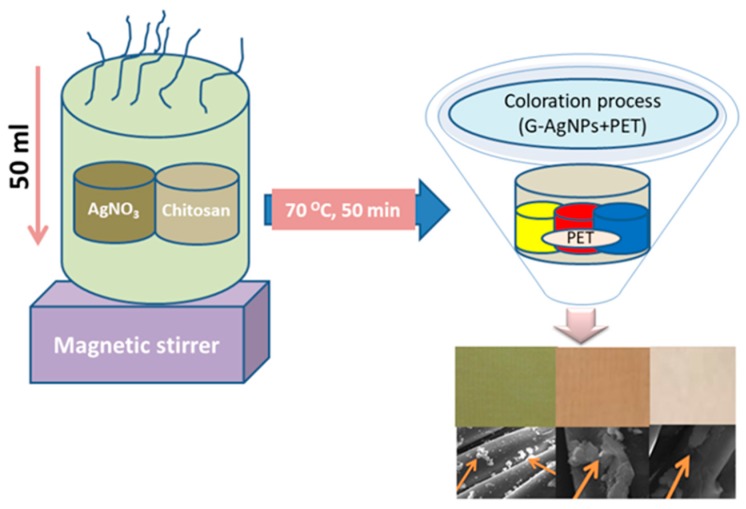
G-AgNPs synthesis and coloration on PET fabric.

Initially, chitosan-mediated green silver nanoparticles (G-AgNPs) were synthesized by adding 1.5 g AgNO_3_ to 0.65 g chitosan in 50 mL solution, with the final solution being magnetically stirred at 70 °C for 50 min. The formation of G-AgNPs was confirmed by observing the change of color solution from colorless to yellow and the sample was termed as yellow colored G-AgNPs solution. Further, by adding 3.5 g AgNO_3_ and 3.3 g AgNO_3_ with 0.5 g ascorbic acid to the same solution, two types of colored AgNPs solution were observed, red and blue, respectively, which were termed as red colored G-AgNPs and blue colored G-AgNPs solutions. For a better synthesis protocol, the solution was further stirred for 90 min at 70 °C. The green coloration of PET fabric through as-prepared G-AgNPs was initiated by immersing the PET (5 g) into the prepared AgNPs solution under ambient conditions. Then, the solution was shaken for 45 min. The liquor ratio of AgNPs solution to fabric was 1:20. The coloration was performed in a laboratory dyeing machine bath at 135 °C temperature for 45 min. After coloration, the fabric was rinsed again and dried at ambient temperature in an oven dryer. Scheme 1 represents the synthesis and coloration procedure. 

### 2.3. Characterization

The UV-Vis extinction spectra were measured using a UV–vis spectrophotometer (Shimadzu, Japan) at 300–600 nm. The morphologies of G-AgNPs were determined by transmission electron microscope (TEM, HT7700, Hitachi High Technologies America, Inc., Schaumburg, IL, USA) operating at 100 kV. The diameter and size distribution of G-AgNPs were calculated by Image J software (National Institutes of Health (NIH), Bethesda, MD, USA) using TEM images. Fourier transform infrared (FT-IR) spectra of G-AgNPs@PET were recorded in KBr pellets using an FT-IR spectrophotometer (Bruker Optics, Ettlingen, Germany) in the wavelength range of 4000–500 cm^−1^. The morphologies of samples were investigated using a scanning electron microscope (SEM, S-4800, Hitachi Ltd., Tokyo, Japan) at 5 kV. Thermal analysis was measured using a thermogravimetric analyzer (TGA V5.1A DUPONT2000). The samples were heated from 50 °C to 600 °C at the rate of 10 °C min^−1^ under the nitrogen (N_2_) atmosphere.

### 2.4. Color Measurement and Color Fastness

The color properties (reflectance curve, K/S and CIE L*a*b* values) of G-AgNPs@PET fabrics were evaluated using a Macbeth Color-Eye7000A spectrophotometer (Gretag Macbeth, Singapore) within the visible spectrum (λ = 400–700 nm) at D65 daylight and 10° standard observer. Wash fastness of the prepared samples was tested according to ISO 105-CO3 (1989) method [32]. Light fastness was tested according to ISO 105-BO2 (1989) method [33]. Dry and wet rubbing fastness of the samples were tested according to ISO 105-X12 (1987) method [34].

### 2.5. Antibacterial Properties

The antibacterial efficiency of G-AgNPs-treated fabrics was initially measured by a disc diffusion assay method (zone of inhibition assay) against *Escherichia coli* (Gram-negative) and *Staphylococcus aureus* (Gram-positive). The quantitative analysis of the antibacterial activity in terms of bacterial reduction percentage was done according to test method GB/T 20944.3-2008 [35]. The laundering durability (repeated washing cycles) of the antibacterial activity of G-AgNPs treated fabrics was also investigated for up to 5 and 10 washing cycles.

## 3. Results and Discussion

### 3.1. UV-Vis Extinction Spectra of G-AgNPs

**Figure 1:**
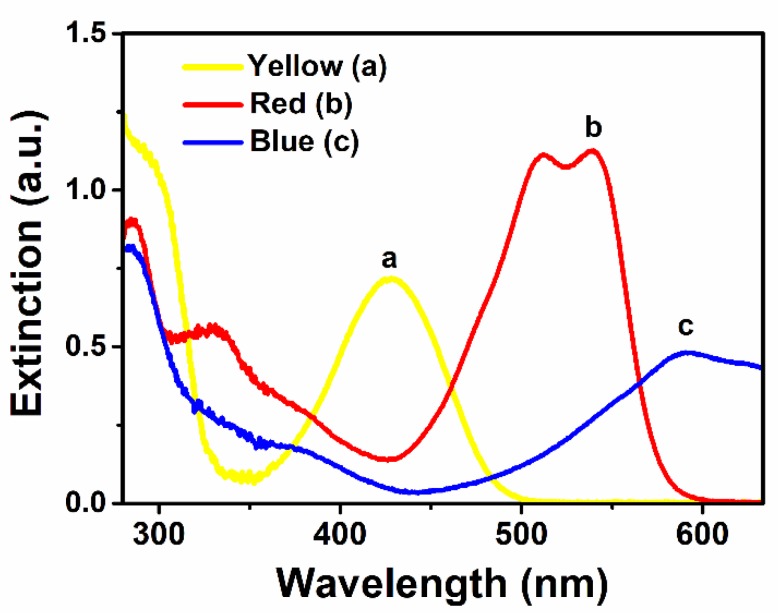
UV-Vis extinction spectra of: (**a**) yellow G-AgNPs; (**b**) red G-AgNPs; and (**c**) blue G-AgNPs.

In general, a solution color change is considered to be a common indicator for the confirmation of metal nanoparticles. Figure 1 presents the UV-Vis extinction spectra of G-AgNPs with three different colors (yellow, red and blue); successful formation of G-AgNPs via Ag^+^ ion reduction was confirmed. The presented three different color extinction spectra were related to yellow, red and blue (Figure 1a–c) and their main extinction bands corresponded to 432, 547 and 597 nm, respectively. This phenomenon may be ascribed to dipole plasmon plane bands of the G-AgNPs. 

### 3.2. TEM Images of G-AgNPs

**Figure 2:**
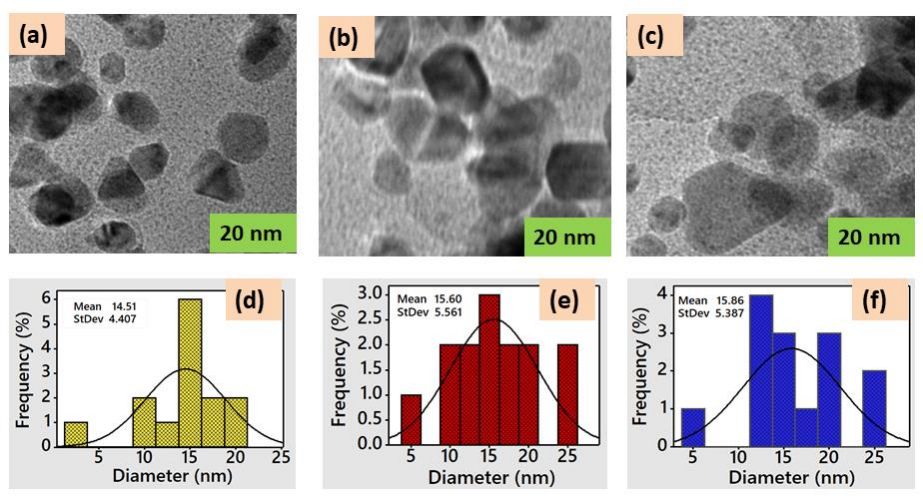
TEM image of: (**a**) yellow G-AgNPs; (**b**) red G-AgNPs; (**c**) blue G-AgNPs; and their respective size distributions (**d**–**f**).

The morphology and size distribution of the G-AgNPs was investigated by TEM image, as shown in Figure 2a–c. In Figure 2a, the TEM image suggests that the morphologies of yellow colored G-AgNPs showed spherical shape with well dispersed. In Figure 2b, in the case of red colored G-AgNPs, the morphology was also spherical but dispersed in sponge form. In Figure 2c, the TEM image of blue colored G-AgNPs reveals that also light spherical shape with reflection dispersed. The average particle size of G-AgNPs observed size distribution in a narrow size. In comparison with the results of three colored G-AgNPs particle size, yellow colored G-AgNPs (Figure 2d) exhibited smaller average particle size (14.51 nm), red colored G-AGNPs (Figure 2e) showed a bit higher average particle size (15.60 nm) and blue colored G-AgNPs demonstrated the biggest average particle size (15.86 nm). It was noticed that the majority of average particles (90%) were positioned between 10 and 20 nm, which is a sign of sufficient homogeneity and good dispersion of G-AgNPs. As a result, it was expected that G-AgNPs treated fabrics would also have well dispersed nanoparticles with high homogeneity, which could be further confirmed by SEM analysis. 

### 3.3. Coloration Process

**Figure 3:**
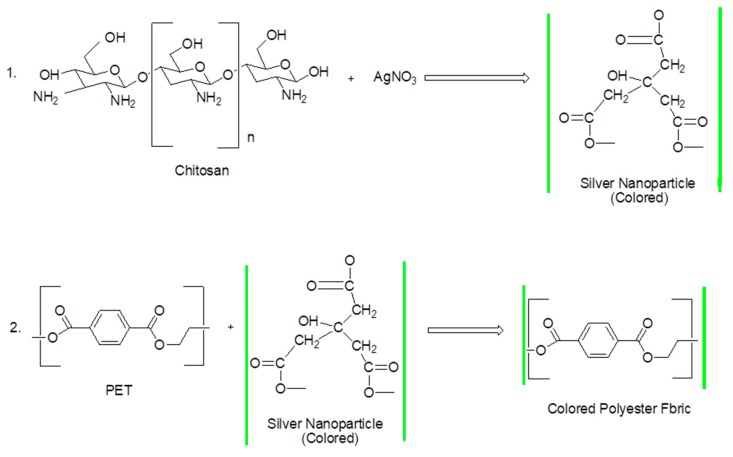
Proposed mechanism of G-AgNPs@PET.

The coloration technique involved the use of a dip coating method. The structural coloration of polyester fabric with green synthesized AgNPs was successfully deposited on the fabric’s surface, and the proposed mechanism is shown in Figure 3. First, due to its non-toxic and biocompatible properties, CS was used as the reducing and stabilizing agent for the green synthesis of G-AgNPs. The chitosan chain contains amino groups that interact with the silver nitrate through stabilization as a favored medium, forming colored green silver nanoparticles (G-AgNPs) (Figure 3). Then, the coloration process was initiated with the addition of three colors of G-AgNPs into the polyester (PET) fabrics in a one-step bath. To facilitate good deposition of G-AgNPs onto the PET fabrics, the latter were first immersed into an aqueous G-AgNPs solution, where Ag^+^ ions aggregate and function as reaction points for developing AgNPs inside the fabric surface. When the temperature was raised to 135 °C, the reducing end groups of PET are proposed to be more ready for reducing Ag^+^ to nanosized Ag^0^ inside the PET structure, subsequently leading to its coloration. The AgNPs stabilization may be associated with the PET chain steric effect as well as rinsing the unbound AgNPs being dispatched from colored fabrics. Finally, the reaction between Ag^+^ ions and functional groups of the fabric surface was terminated by precipitation of silver in the solution (Equations (1) and (2)).

(1) $$CS+Ag^{+}\overset{T\;=\;70{}^{\circ}C}{\underset{50\;\min}{\to }}{\left[Ag/CS\right]}^{+}$$

(2) $${\left[Ag/CS/PET\right]}^{+}\overset{T\;=\;135{}^{\circ}C}{\underset{45\;\min}{\to }}{\left[Ag/CS/PET\right]}^{+}↓$$

**Figure 4:**
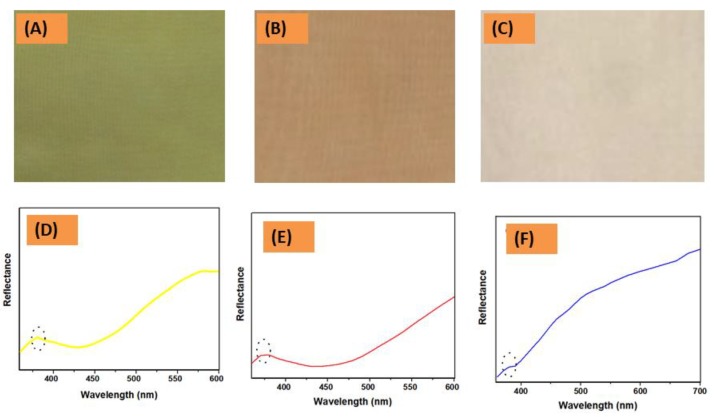
(**A**–**C**) Photographs; and (**D**–**F**) reflectance curves of the colored fabric.

Figure 4A–C illustrates the yellow- (YCF), red- (RCF) and blue-colored fabrics (BCF), respectively. It was found that colors of the G-AgNPs@PET fabrics were darker than the original solution of G-AgNPs, a result which may be attributed to the encompassing of the G-AgNPs [36]. In addition, the reflectance spectra of G-AgNPs@PET fabrics were investigated to better understand their color properties. As shown in Figure 4D–F, the reflectance bands corresponded to YCF (372 nm), RCF (352 nm) and BCF (368 nm). It was observed that reflectance bands varied according to their color characteristics. However, it can be assumed that the G-AgNPs on the PET fabric surface were deposited with higher hydrophobic environment than water solvation.

### 3.4. Color Measurement and Fastness Properties

Colorimetric data were measured for G-AgNPs@PET to study the effect of G-AgNPs on the color properties (Table 1). YCF demonstrated decreased L* values with higher b* than RCF and BCF, indicating that the color became darker and more saturated in the case of YCF. On the other hand, redness (a* values) was comparatively higher for RCF and BCF compared to YCF, most likely due to the presence of a reddish tone on the fabric surface. As expected, the color strength (K/S) values were higher for YCF than RCF and BCF, a result support by the decreased L* values.

The color fastness (washing, rubbing and light fastness) is related to the color-fading nature of fabric, a property which is a crucial factor for commercial end uses. The color-fastness properties of G-AgNPs@PET fabrics were determined (Table 2). With respect to washing fastness, all colored fabrics showed good fastness ratings in terms of demonstrating little color change (rating above 4). The light fastness of fabrics was acquired with a rating above 5, which is also satisfactory. The rubbing fastness (wet and dry) of colored fabrics was achieved in the acceptable range. Overall, all of the above results were determined to be acceptable according to the color-fastness rating of the Chinese National Standards for Textiles [25].

### 3.5. FT-IR Analysis

**Figure 5:**
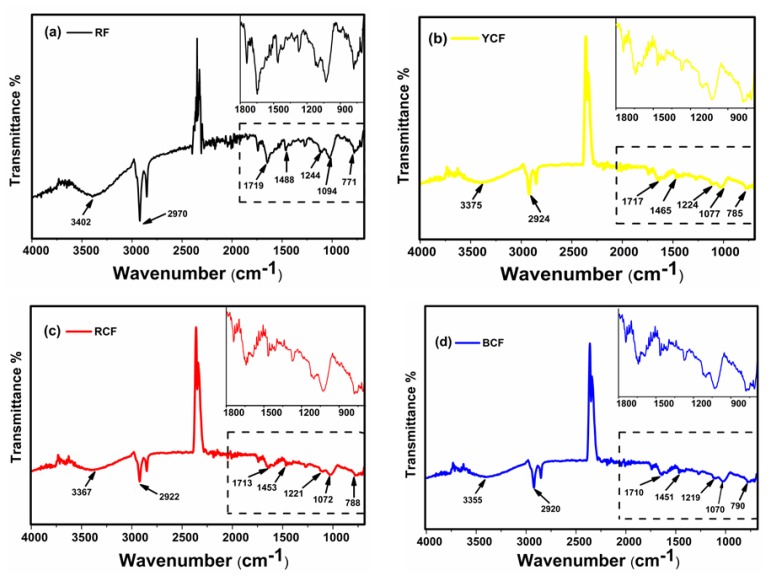
FT-IR spectra of: (**a**) RF; (**b**) YCF; (**c**) RCF; and (**d**) BCF.

Fourier transform infrared spectroscopy (FTIR) was used to investigate the presence of functional groups of raw and G-AgNPs-treated PET fabric (Figure 5a–d). For the spectrum of RF (Figure 5a), the peaks at 3402 and 2970 cm^−1^ were assigned to intermolecular O–H bonded to C=O groups and C–H stretching in the polyester chain [37]. The peak at 1719 cm^−1^ was ascribed to the C=O group of aromatic ester linkage, while the peaks at 1244 and 1094 cm^−1^ can be assigned to C=O ester bond stretching vibration [38]. The small peak at 1488 cm^−1^ was associated with C=C stretching vibration and the end characteristic band at 771 cm^−1^ corresponded to in-plane C–O stretching vibration. As shown in Figure 5b–d, after G-AgNPs treatment, the intensity of the bands at 3000–3500 cm^−1^ region increased and widened compared to RF. The band at 1719 cm^−1^ was shifted to 1717, 1713 and 1711 cm^−1^ (Figure 5b–d, respectively) for polyester and another band at 771 cm^−1^ also shifted to 785, 788 and 790 cm^−1^ (Figure 5b–d, respectively), results associated with C–H bending vibrations of benzene rings in the polyester due to incorporation of the G-AgNPs on the PET surface. These results indicated that there were some interactions between the G-AgNPs and PET.

### 3.6. TGA Analysis

**Figure 6:**
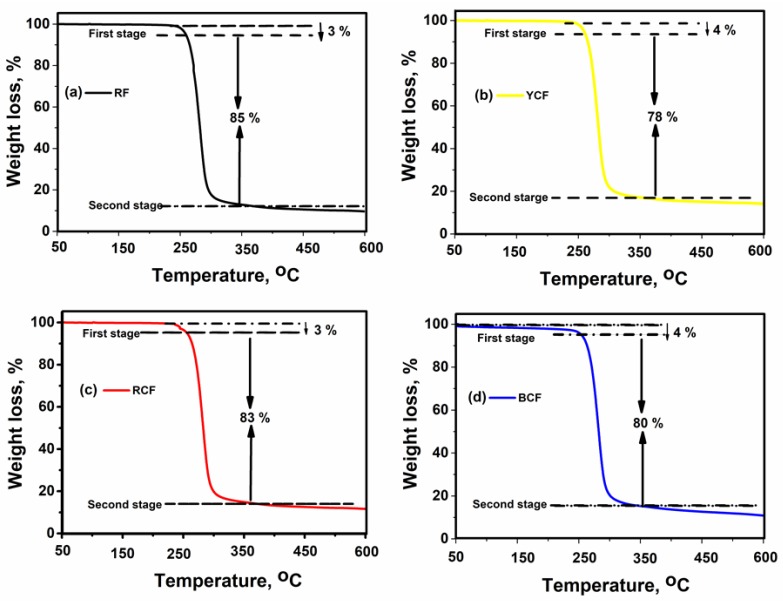
TGA curves of: (**a**) RF; (**b**) YCF; (**c**) RCF; and (**d**) BCF.

Thermal measurements of raw and G-AgNPs-treated PET fabrics were determined using thermogravimetric analysis (TGA) (Figure 6a–d). Figure 6a shows the thermogram of RF. It was observed that decomposition of RF was in two stages; the initial stage involved 3% weight loss within 10–250 °C due to moisture absorption of the fabric. The second stage was ascribed to the most weight loss that was observed within 278–350 °C due to depolymerization of the polyester. Figure 6b shows a TGA curve for the YCF sample; the two stages of degradation behavior also occurred but shifted to a higher temperature range compared to RF. Maximum weight loss was observed in the second stage within 288–382 °C. Figure 6c illustrates the TGA curve for the RCF sample; although a similar initial 3% weight loss stage to the RF was observed, in the second stage, a higher degradation temperature of 268–358 °C was acquired compared to RF. Figure 6d shows the TGA curve for BCF, with the same thermogram but a higher degradation at the initial temperature (4%) and second stage at 266–356 °C. From the curve, it can be seen that incorporation of G-AgNPs on the PET fabric surface increased its thermal stability, with YCF demonstrating the highest thermal stability. The TGA curve provides confirmation that our experimental samples have good flame-retardant protection properties. 

### 3.7. SEM Analysis

**Figure 7:**
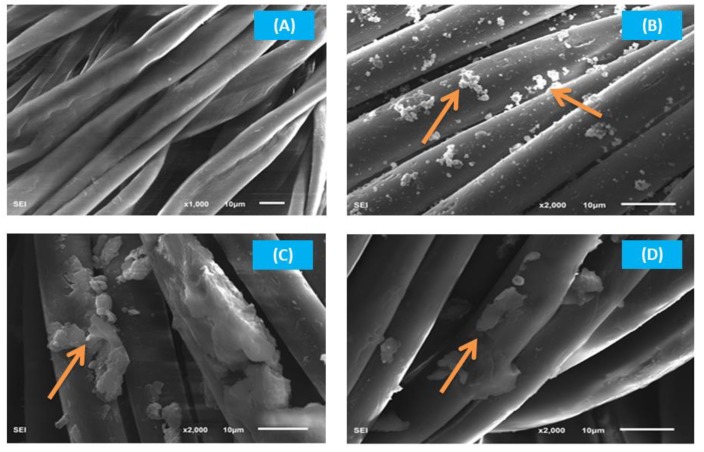
SEM images of: (**A**) RF; (**B**), YCF; (**C**) RCF; and (**D**) BCF.

The surface morphologies of raw and G-AgNPs-deposited PET samples were investigated using SEM, as shown in Figure 7. The plain smooth fibrous surface of the raw fabric (RF) was observed (Figure 7A), with a regular morphology of PET fibers [39]. Figure 7B–D shows the morphology of G-AgNPs-treated PET fabrics. It is clearly seen in the images that nanoparticles were deposited on the fabric surface, increasing the coloration properties of the fabric. For YCF (Figure 7B), the nanoparticles were homogenously distributed and received the highest color strength values (Table 1), while RCF and BCF showed uncontrolled dispersed nanoparticles on their surfaces (Figure 7C,D). These results confirm that the strong metal binding ability and adhesion properties of chitosan may have increased the activity of silver nanoparticles [40]. 

### 3.8. Antibacterial Properties 

**Figure 8:**
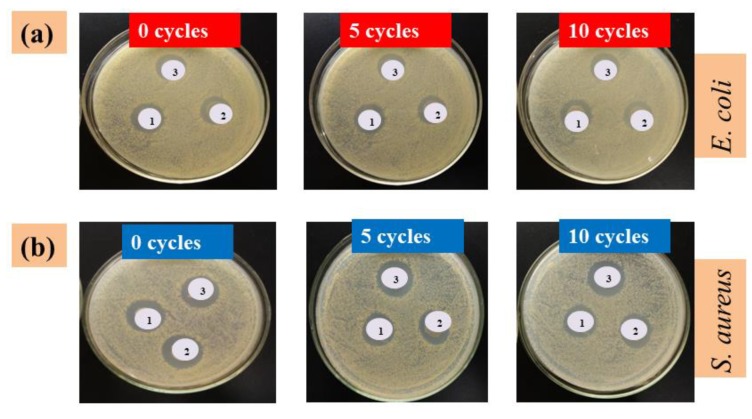
Inhibition zone photographs of YCF, RCF, and BCF before (0 cycles) and after 5 and 10 washing cycles against bacteria: (**a**) *E. coli*; and (**b**) *S. aureus*.

The antibacterial property of PET-based fabrics is considered to be a critical indicator for functional applications in terms of non-antibacterial properties of neat PET-based fabrics. In recent years, several antibacterial agents have been applied for improving their bacterial inhibition growth abilities [41]. Silver nanoparticles (AgNPs) have been regarded as emerging antibacterial agents with unique properties [42]. Figure 8a,b illustrates inhibition zone test results of three colored G-AgNPs-PET fabrics (YCF, RCF, and BCF), which were all evaluated before (0 cycles) and after 5 and 10 washing cycles against *Escherichia coli* (Gram-negative) and *Staphylococcus aureus* (Gram-positive), respectively. Clear zones of bacterial growth inhibition were noted around all G-AgNPs-treated fabrics. These fabrics prior to washing retained more G-AgNPs with better inhibition zone diameters, and demonstrated high bacterial reduction capabilities. 

**Figure 9:**
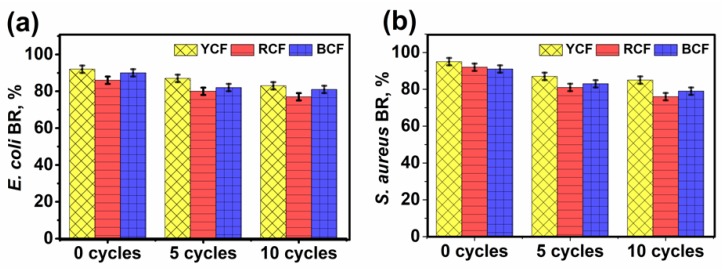
Bacterial reduction (%) of YCF, RCF, and BCF before (0 cycles) and after 5 and 10 washing cycles against bacteria: (**a**) *E. coli*; and (**b**) *S. aureus*.

Furthermore, the quantitative assessment of G-AgNPs@PET fabric antibacterial activity was observed before (0 cycles) and after 5 and 10 washing cycles according to the bacterial reduction percentage method (AATCC 100) (Figure 9a,b). It was observed that G-AgNPs-treated fabrics exhibited demonstrable antibacterial properties and, even after ten washing cycles, they were able to remove more than 80% of bacteria. Among all the samples, YCF demonstrated better antibacterial activity in terms of large inhibition zone diameters and higher bacterial reduction percentages against both Gram-positive and -negative bacterial strains. This result was achieved due to the biocidal action of the treated fabric due to Ag^+^ ion leaching [43]. Samrot et al. [44] also reported that the yellow color is associated with high antibacterial activity.

## 4. Conclusions

A simple and feasible method is presented in this work to produce simultaneous colorful and multifunctional polyester fabric using green silver nanoparticles (G-AgNPs@PET). The synthesis of G-AgNPs was confirmed via UV-visible, TEM, SEM and FT-IR measurements determining the successful deposition of G-AgNPs on the PET fabric surface as well as the thermal properties of PET fabric being enhanced after addition of G-AgNPs. The coloration and fastness properties were also improved with a bacterial reduction rate above 90% for both bacterial strains. The presented method may provide a potential novel coloration process for use in the textile industry with desirable functional properties.

## Figures and Tables

**Table 1:** Color measurements and *K/S* values.

Samples	L*	a*	b*	K/S
YCF	59.57	9.71	27.34	3.84
RCF	59.71	10.39	20.72	2.33
BCF	64.39	12.31	17.21	1.22

**Table 2:** Fastness properties of the colored fabrics.

Samples	WF	LF	RF	
			wet	dry
YCF	4–5	5–6	4	4–5
RCF	4	5	3–4	4
BCF	4	5	4	4

WF, Wash Fastness; LF, Light Fastness; RF, Rubbing Fastness.
